# MXene-Based Porous Monoliths

**DOI:** 10.3390/nano12213792

**Published:** 2022-10-27

**Authors:** Yang Yang, Kaijuan Li, Yaxin Wang, Zhanpeng Wu, Thomas P. Russell, Shaowei Shi

**Affiliations:** 1 Beijing Advanced Innovation Center for Soft Matter Science and Engineering, State Key Laboratory of Organic-Inorganic Composites, Beijing University of Chemical Technology, Beijing 100029, China; 2 Department of Polymer Science and Engineering, University of Massachusetts, Amherst, MA 01003, USA; 3 Materials Sciences Division, Lawrence Berkeley National Laboratory, Berkeley, CA 94720, USA; 4 Beijing Engineering Research Center for the Synthesis and Applications of Waterborne Polymers, Beijing University of Chemical Technology, Beijing 100029, China

**Keywords:** 2D nanomaterials, MXenes, porous architecture, assembly

## Abstract

In the past decade, a thriving family of 2D nanomaterials, transition-metal carbides/nitrides (MXenes), have garnered tremendous interest due to its intriguing physical/chemical properties, structural features, and versatile functionality. Integrating these 2D nanosheets into 3D monoliths offers an exciting and powerful platform for translating their fundamental advantages into practical applications. Introducing internal pores, such as isotropic pores and aligned channels, within the monoliths can not only address the restacking of MXenes, but also afford a series of novel and, in some cases, unique structural merits to advance the utility of the MXene-based materials. Here, a brief overview of the development of MXene-based porous monoliths, in terms of the types of microstructures, is provided, focusing on the pore design and how the porous microstructure affects the application performance.

## 1. Introduction

Transition-metal carbides/nitrides (MXenes) are a thriving family of 2D nanomaterials that have attracted tremendous attention since their discovery in 2011 [1]. Generically, MXenes have a chemical formula of M*_n+1_*X*_n_*T*_x_*, where M, X, and T*_x_* represent the transition metal site, carbon and/or nitrogen, and surface terminations of O/OH/F, respectively, and *n* equals to 1–4 [2,3]. They have a structure consisting of two or more layers of transition metal atoms packed into a 2D lattice that are intervened by carbon and/or nitrogen layers occupying the octahedral sites between the adjacent transition metal layers, with multiple surface terminations on the surface of the outer transition metal layers [4]. MXenes with two transition metal elements occupying M sites possess two forms that are the solid solution form and ordered form. The former form represents a random distribution of two metals in the M layers, showing a chemical formula of (M′,M″)*_n+1_*X*_n_*T*_x_* (M′ and M′′ represent two different metals) [5,6,7]. The latter one can be further divided into in-plane ordering, where M′ and M′′ atoms generate alternating chains within the same M layer (termed as *i*-MXenes) [8,9], and out-of-plane ordering, where M′′ atoms form the inner M layers while M′ atoms are placed in the outer layers (termed as *o*-MXenes) [10,11]. Moreover, a kind of high-entropy MXene with four transition metals, TiVNbMoC_3_T*_x_* and TiVCrMoC_3_T*_x_*, also was synthesized recently [12]. To date, more than 30 MXene members have been experimentally made, and over 100 have been theoretically predicted. Such a large family with sufficient, tunable composition, structure, and surface/interlayer chemistry has shown useful and versatile properties, e.g., high electrical conductivity [13], high internal light-to-heat conversion efficiency [14], superior hydrophilicity, and notable mechanical property [15,16,17,18], leading to promising potential in energy storage [18,19,20] and harvesting [21], electromagnetic interference (EMI) shielding [22,23], sensing [24], catalysis [25,26,27,28], etc.

MXenes are produced by selectively etching the A-layer atoms (such as Al, Si, and Ga) of MAX phases [29]. This top-down process can be scaled up, not limited by the scalability issue like with bottom-up synthesis adopted by some 2D materials, allowing MXenes the potential to transition from laboratory use to industrial production [30]. However, when integrating MXenes into macroscopic architectures for practical utilization, they always suffer from inevitable restacking and aggregation due to the van der Waals forces, impeding the accessibility of surface-active sites, reducing the elasticity/mechanical strength, and limiting the effective loading of other functional materials [31,32,33,34]. The close packing results in a large wave and mass transport resistance [35,36] that have become the major challenge for the development of MXene-based functional devices.

Viewing from the success of the integration of graphene (GO), two effective strategies, including adding interlayer spacers and introducing internal pores into the constructs, can address the restacking issue [37,38,39,40]. In 2017, Gogotsi and co-workers made a significant step forward by applying two distinct designs for Ti_3_C_2_T*_x_* MXene electrodes, where one is noted as a hydrogel film with water molecules serving as interlayer spacers, while the other film has a 3D macroporous structure [41]. Both electrodes show improved ion accessibility to redox-active sites, delivering exceptional capacitance performance, and opening new opportunities for energy harvesting and storage. Yet, a smaller ion transport resistance was presented in the macroporous film, displaying the potential of porous microstructure in some applications required for fast mass transport. Since then, porous MXene architectures have ushered in rapid development.

**Figure 1:**
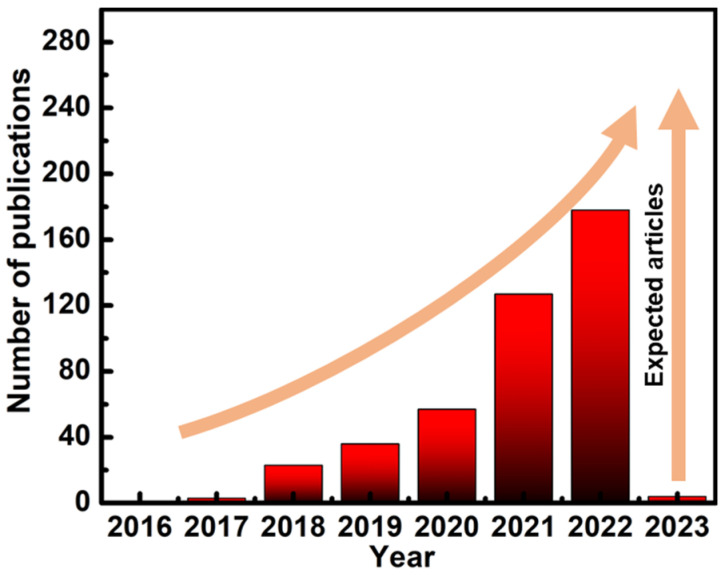
Growing importance of MXene-based porous monoliths in increasing number of SCI indexed publications (source: SciFinder 2022).

Macroscopic porous monoliths, such as aerogels with very high porosities, provide scaffolding with exceptionally high surface-to-volume ratios that can support 2D materials, giving a superior strategy for the integration of MXenes, evidenced by the following merits: (1) the aggregation of MXenes can be reduced, even eliminated, in the matrix; (2) the artificial internal pores facilitate the wave/mass transport, and the high specific area enables easy access to MXenes; and (3) the inner pores and pore walls can, in turn, serve as scaffolding to support functional materials, engineering multilevel hierarchical structures for a variety of applications. In the past several years, advanced MXene aerogels, hydrogels, and foams have garnered much interest and have been used for energy conversion and storage, EMI shielding/electromagnetic wave (EMW) absorption, wearable piezoresistive sensors, and water steam generator/solar water desalination [42,43,44,45,46,47], with a rapid growing number of related research publications (Figure 1).

An emerging aspect of MXene-based porous monoliths is to design and tailor the structure of pores to meet the requirement of application. Growing attention is being drawn into the multiscale design of the microstructure, ranging from geometries, such as morphology (pore, channel, and lamella), pore size/channel spacing, and openings throughout the walls, to regularity (isotropic, local oriented, and long-range ordered configuration). Particularly, a type of aligned porous microstructure with oriented walls and channels is of great potential. In 2018, Yang and co-workers firstly achieved the vertical alignment of MXene by applying a uniaxial in-plane mechanical shear force to the discotic lamellar liquid crystal phase of Ti_3_C_2_T*_x_* [48]. A 2D macroscopic film with highly ordered, vertically aligned structure was then fabricated. The unique microstructure imparts the film with a shorter ion transport path and a larger number of available active sites than the stacked MXene films. This aligned film shows an excellent thickness-independent electrochemical performance when using as supercapacitor electrodes. Very closely, Zhang and co-workers demonstrated remarkable EMI-shielding performance in a Ti_3_C_2_T*_x_*-based hybrid 3D aerogel with an aligned structure [49]. It was found that the ingenious design of the microstructure can not only enhance the instinct performance of MXene monoliths, but also benefit it by exploring new applications, e.g., solar seawater desalination [50]. Such state-of-the-art progress and achievements are highly expected to be summarized and discussed to inspire the development of the next generation of MXene-based materials and devices.

**Figure 2:**
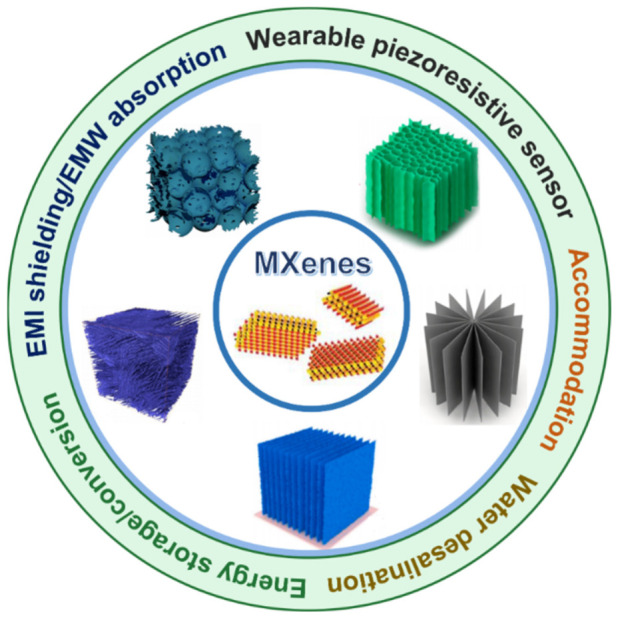
MXene-based porous monoliths: types and applications. Reproduced with permission from ref. [51]. Copyright 2016, Wiley-VCH. Reproduced with permission from ref. [52]. Copyright 2019, Elsevier. Reproduced with permission from ref. [53]. Copyright 2022, American Chemical Society. Reproduced with permission from ref. [54]. Copyright 2020, Elsevier. Reproduced with permission from ref. [55]. Copyright 2022, American Chemical Society.

Here, we review the recent progress on the MXene-based porous monoliths, where the different types of microstructures and the structure–property–application affinity are examined (Figure 2). Finally, opportunities that lie ahead in this emerging field are discussed.

## 2. Types of Microstructures and Fabrication of MXene-based Porous Monoliths

**Figure 3:**
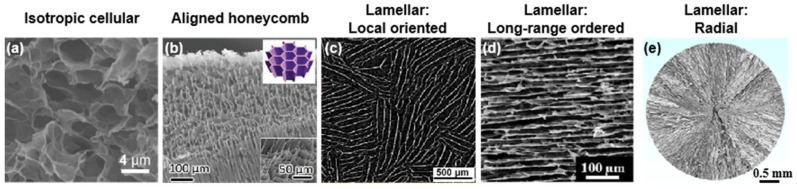
Five typical microstructures in MXene-based porous monoliths: (**a**) isotropic cellular, (**b**) aligned honeycomb, (**c**) local oriented lamellar, (**d**) long-range ordered lamellar, and (**e**) radial lamellar structures. (**a**) is reproduced with permission from ref. [34]. Copyright 2018, American Chemical Society. (**b**) is reproduced with permission from ref. [59]. Copyright 2020, Wiley-VCH. (**c**) is reproduced with permission from ref. [55]. Copyright 2022, American Chemical Society. (**d**) is reproduced with permission from ref. [60]. Copyright 2019, American Chemical Society. (**e**) is reproduced with permission from ref. [53]. Copyright 2022, American Chemical Society.

Porous monoliths discussed in this review are designated by 3D macroscopic architectures with a very large volume ratio of internal pores. Taking aerogel as an example, it possesses a solid skeleton with porosity over 90%, seen as the least dense material [56,57,58]. Since the morphology and regularity of pores play significant roles in the property and function of monoliths, classifying the microstructures is of interest. There are five typical types of microstructures, including isotropic cellular, aligned honeycomb, and aligned lamellar configurations for which the last can be further divided into local oriented, long-range ordered, and radial ones. To date, all of them have been introduced into the MXene monoliths (Figure 3) [34,53,55,59,60]. Moreover, by adjusting the interactions between the components (MXenes and additives), or by varying the manufacturing parameters (concentrations, and freezing rate/direction), a high degree control of the microstructure over the MXene monoliths has been achieved, enabling a wide range of hierarchies at the nano-, micro-, and mesoscales.

### 2.1. Isotropic Cellular Structure

Porous monoliths with isotropic cellular microstructure feature an interconnected framework, where the pores have no preferential orientation. The building blocks in cell walls are closely packed, while individual cells range from nanoscopic- to microscopic-length scales. MXene monoliths, having isotropic pores, are easily produced and can be achieved by network pre-assembly followed by the solidification process, where the pre-assembly can be realized by either an assisted gelation or a melding of emulsion droplets.

#### 2.1.1. Assisted Gelation

Gelation is a commonly used method to integrate nano-building blocks into 3D monoliths, but is hardly adopted to MXenes on their own, due to the aggregating and stacking tendency of MXenes. The superior hydrophilicity imparted by the surface groups further challenges their assembly in an aqueous environment [18,31]. Introducing a second component as crosslinkers or gelators is a simple strategy to counterbalance the hydrophilicity of MXenes to generate 3D assemblies. In general, the continuous phase in MXene gel is confined within a 3D structured network that is physically and/or chemically crosslinked by gelators, which can be GO, polymers, metal cations, or a combination of these.

**Figure 4:**
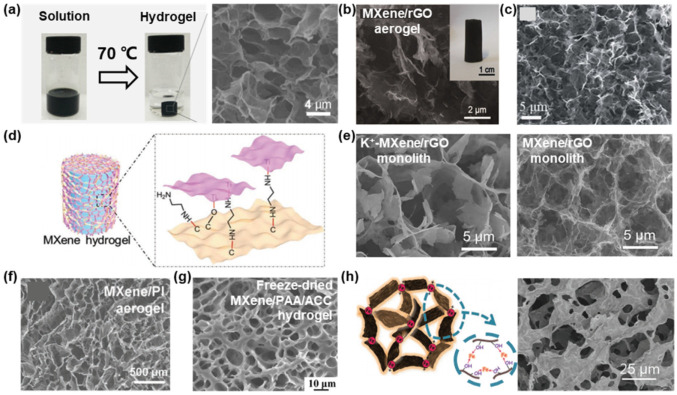
Digital photographs and SEM images of MXene/rGO (**a**) hydrogel and (**b**,**c**) aerogels with isotropic cellular microstructure. (**d**) Schematic illustration of EDA-MXene/rGO hydrogel. (**e**) SEM images of K^+^-MXene/rGO monolith and MXene/rGO monolith. (**f**) SEM image of MXene/PI aerogel. (**g**) SEM image of freeze-dried MXene/PAA/amorphous calcium carbonate (ACC) hydrogel. (**h**) Schematic illustration and SEM image of Fe^2+^-MXene monolith. (**a**) is reproduced with permission from ref. [34]. Copyright 2018, American Chemical Society. (**b**) is reproduced with permission from ref. [63]. Copyright 2018, Wiley-VCH. (**c**) is reproduced with permission from ref. [64]. Copyright 2019, The Royal Society of Chemistry. (**d**) is reproduced with permission from ref. [67]. Copyright 2019, Wiley-VCH. (**e**) is reproduced with permission from ref. [69]. Copyright 2021, Elsevier. (**f**) is reproduced with permission from ref. [33]. Copyright 2018, Wiley-VCH. (**g**) is reproduced with permission from ref. [79]. Copyright 2021, American Chemical Society. (**h**) is reproduced with permission from ref. [81]. Copyright 2019, Wiley-VCH.

The tailorable hydrophilic–hydrophobic balance of GO-reduced GO (rGO) endows them with an extraordinary gelation ability [40], enabling MXene nanosheets to be integrated into their 3D framework. Xu and co-workers showed that, by heating a mixture of Ti_3_C_2_T*_x_* and GO, the Ti_3_C_2_T*_x_* partially remove the oxygen-containing surface species on GO by a valence transfer, reducing GO to rGO, and increasing the hydrophobicity and π-conjugated structures of rGO [34]. rGO then assemble into a 3D porous framework, while Ti_3_C_2_T*_x_* are self-converged and incorporated into this framework driven by hydrogen bonding, thereby generating a Ti_3_C_2_T*_x_*/rGO hydrogel with an interconnected network with open pores and thin walls (Figure 4a). It is notable that a TiO_2_ phase always appears in the prepared hydrogel, probably ascribing to the oxidation of Ti_3_C_2_T*_x_*. MXenes are susceptible to oxidation when stored in water at room temperature [61,62]. Generally, the oxidation starts from the edges, causing the degradation of the MXene structure and the formation of metal-oxide nanocrystals, then develops through nucleation and growth throughout the entire surface, which will impede the electrical conductivity [18,61]. To overcome this issue, a reducing agent, NaHSO_3_, was introduced in the preparation procedure of Ti_3_C_2_T*_x_*/rGO hydrogel. No TiO_2_ phase was observed in the resultant hydrogel, indicating that the oxidation of Ti_3_C_2_T*_x_* was effectively suppressed. Afterward, using the hydrogels as precursors, MXene/rGO aerogels can be easily fabricated after freeze drying [63,64,65,66]. SEM images show that the microstructure of aerogel is akin to the precursor with a pore size range at the micron level (Figure 4b,c). By introducing interfacial mediators into MXene and rGO, the structure of the walls within the hybrid monoliths can be finely engineered. For instance, when using small molecules, such as ethylenediamine (EDA) [67] or amino-propyltriethoxysilane [68], as interlayer spacers to bond the rGO layers and Ti_3_C_2_T*_x_* nanosheets (Figure 4d), the accessible surface area of the porous monoliths will increase. On the other hand, Yang and co-workers reported that adding a monovalent cation, K^+^, can weaken the repulsive electrostatic interactions, promoting a “face-to-face” stacking of Ti_3_C_2_T*_x_* nanosheets, resulting in a thicker laminated wall (Figure 4e) [69].

MXene can uniformly disperse in a polymer matrix to form a hybrid monolith with isotropic pores. The driving force can be various physical/chemical interactions, including polymer chain entanglements, ionic interactions, and hydrogen and/or covalent bonding. Details of the gelation mechanism and fabrication process were summarized previously [45]. Since the report of the Ti_3_C_2_T*_x_*/poly(vinyl alcohol) (PVA) hybrid hydrogel [70], a series of polymers have been selected as the support to generate MXene-based hydrogels, aerogels, and organohydrogels [71,72,73,74,75,76,77,78,79,80]. For instance, Zhang and co-workers modulated a mixture containing Ti_3_C_2_T*_x_* and poly(amic acid) [33]. After freezing and freeze drying, a robust 3D architecture was formed, driven by the strong interaction between the two components. A thermal annealing was then performed to induce the polymerization of poly(amic acid) to generate polyimide (PI) macromolecules, resulting in the final Ti_3_C_2_T*_x_*/PI aerogel with a compact interface and interconnected porous structure (Figure 4f). Recently, Wan and co-workers reported a facile and mild procedure to prepare a Ti_3_C_2_T*_x_*/poly(acrylic acid) (PAA)/amorphous calcium carbonate (ACC) hybrid hydrogel [79]. By mixing the Ti_3_C_2_T*_x_*, PAA, and CaCl_2_ in an aqueous solution then adding Na_2_CO_3_, the MXene-based hydrogel was produced by the synergistic interactions among the surface terminations of Ti_3_C_2_T*_x_* and the carboxylic groups of PAA and Ca^2+^. Figure 4g shows the porous structure of the hydrogel after freeze drying.

The main challenges of MXene gelation in an aqueous environment are their hydrophilicity and strong electrostatic repulsion between the nanosheets, which can be addressed by the addition of metal cations. Divalent metallic ions, e.g., Fe^2+^, Mg^2+^, Co^2+^, and Ni^2+^, have been used to initiate the fast gelation of Ti_3_C_2_T*_x_* in aqueous suspension [81]. The metal ions electrically interact with the surficial groups of Ti_3_C_2_T*_x_*, destroying the electrostatic repulsive force between Ti_3_C_2_T*_x_*, and acting as crosslinkers to lead a phase separation of Ti_3_C_2_T*_x_* from the solution to initiate the gelation, finally forming a 3D porous network (Figure 4h).

#### 2.1.2. Emulsion Template

The liquid–liquid interface provides a versatile platform for integrating nano-building blocks into hierarchical constructs for physical, chemical, and biological applications [82,83,84,85]. Pristine MXene nanosheets are electronegative and hydrophilic, both detrimental for adsorption to the inherently negative oil/water interface. This challenge was addressed by tuning the wetting behavior of Ti_3_C_2_-MXene with a surface modification with cetyl trimethylammonium bromide (CTAB) [86]. Using CTAB-modified Ti_3_C_2_ as emulsifiers, Huang and co-workers produced high internal phase Pickering emulsions as a robust template. After polymerizing the continuous phase, 3D porous materials with a cellular microstructure were achieved.

**Figure 5:**
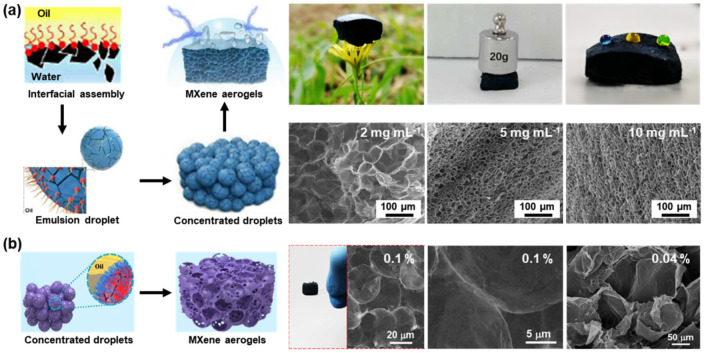
(**a**) Schematic illustration of collaborative assembly of MXSs at toluene–water interface and the MXS aerogel; optical photographs showing the lightweight, robust, and hydrophobic MXS aerogel; and SEM images MXS aerogels prepared from emulsion templates with different MXene concentrations. (**b**) Schematic illustration of JMN aerogel; optical photograph showing the lightweight property; and SEM images of JMN aerogels prepared from emulsion templates with different JMN contents. (**a**) is reproduced with permission from ref. [93]. Copyright 2019, Wiley-VCH. (**b**) is reproduced with permission from ref. [96]. Copyright 2022, Elsevier.

A more convenient strategy, though, is based on the electrostatic interactions between functionalized nanoparticles and organic ligands having a complementary functionality at the liquid–liquid interface to form nanoparticle surfactants (NPSs) that assemble at and are irreversibly bound to the interface [87]. The binding of the NPSs is sufficiently strong so that when the NPS assemblies are compressed to reduce the interfacial area, the assemblies will jam, locking any further shape changes of the liquids, shaping the liquid domains. Such structured liquids afford a simple route to generate hierarchical constructs [88,89,90,91,92]. Using this strategy, Russell and co-workers introduced a MXene-surfactant (MXS), a nanosheet surfactant, with exceptional surface activity by the interfacial interactions between MXene, dispersed in the aqueous phase, and oil-soluble amine-functionalized polyhedral oligomeric silsesquioxane (POSS-NH_2_) *in situ* at the toluene–water interface (Figure 5a) [93]. By homogenizing an aqueous dispersion of Ti_3_C_2_T*_x_* and with a solution of POSS-NH_2_ in toluene, very stable emulsions were formed, even under ultra-centrifugation, allowing them to be concentrated and used as scaffolds and templates. Freeze drying the emulsions leads to a lightweight MXS-based aerogel with an interconnected porous framework and excellent mechanical strength under compression. Since the ligands anchored to the Ti_3_C_2_T*_x_* are hydrophobic, a hydrophobic MXene-based aerogel was produced. Similar types of assemblies could be achieved at the silicone oil–water interface, enabling the 3D printing of MXene all-liquid devices [94]. In addition to the oil–water interface, this interfacial co-assembly method can be performed in an ionic liquid–water biphasic system, underscoring the generality of the liquid–liquid interface in the generation of MXene-based porous monoliths [95].

Polymer chains can also be anchored to the MXene surface directly, where the adsorbed polymer, for example, polystyrene, can be used for its inherent hydrophobic character to generate Janus Ti_3_C_2_T*_x_*-MXene nanofilm (JMN), which can serve as a distinct building block to form Pickering emulsions (Figure 5b) [96]. Here, the densely packed JMNs readily form a continuous network due to the strong inter-sheet interactions and, ultimately, to a lightweight and isotropic JMN-based aerogel. SEM images show the continuous, cellular pores, and the inter-sheet connections, giving direct insights into the porous micromorphology and the integrity of cell walls.

It is worth noting that, in these interfacial assembly systems, the pore size of the aerogels is commensurate with the emulsion droplets, allowing a tailorable microstructure. It was demonstrated that the size of MXS emulsion droplets could be controlled from several tens to hundreds of microns by changing the concentration of Ti_3_C_2_T*_x_*. The resultant MXS aerogels show a tunable pore size from 60 to 15 μm. Similarly, by adjusting the JMN content, pores ranging from 50 to 20 microns can be obtained. As the concentration of JMNs decreases, pores connecting adjacent cells in the porous monolith emerge, due to the incomplete coverage of the liquid domains in the original emulsion.

### 2.2. Aligned Honeycomb Structure

MXene monoliths with an aligned honeycomb structure consist of interconnected and long tubules, where the walls are made of closely packed MXenes. This anisotropic morphology presents a honeycomb-like cellular structure in the transverse plane and a long tubular structure in the longitudinal plane. By using the unidirectional freezing technique or 3D printing, MXene-based porous monoliths with this type of microstructure can be produced.

#### 2.2.1. Unidirectional Freezing

**Figure 6:**
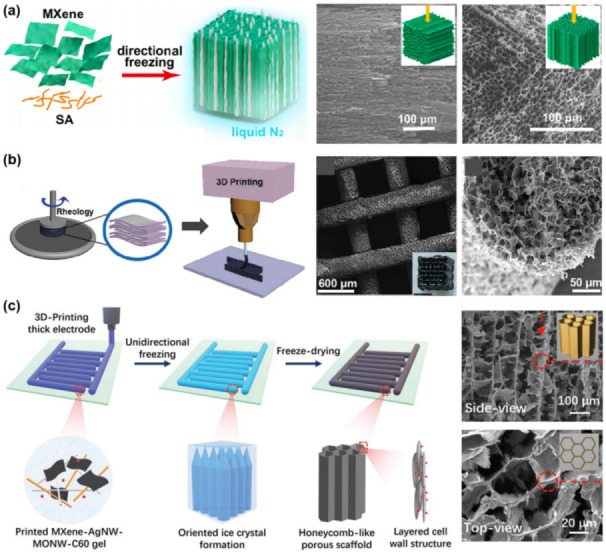
(**a**) Schematic illustration of MXene/SA aerogel with aligned honeycomb microstructure prepared by UFC, and SEM images captured from the side view and top view. (**b**) Schematic illustration showing the 3D-printing MXene architecture, and SEM images showing the MXene architecture and the cross-section of one printed filament. (**c**) Schematic illustration of the MXene architecture prepared by 3D printing and UFC, and SEM images captured from the side view and top view. (**a**) is reproduced with permission from ref. [52]. Copyright 2019, Elsevier. (**b**) is reproduced with permission from ref. [103]. Copyright 2019, Wiley-VCH. (**c**) is reproduced with permission from ref. [109]. Copyright 2020, Wiley-VCH.

Directional freezing, also termed freeze casting (FC), has been well developed to fabricate hierarchical porous materials [97,98,99]. By confining the heat reduction along the axial direction, e.g.*,* from bottom to top, ice crystals nucleate on the cooled bottom surface and propagate along the temperature gradient, offering an aligned ice-template to concentrate and squeeze the building blocks into the gaps between the crystal boundaries, yielding highly ordered constructs. Yu and co-workers firstly reported a Ti_3_C_2_T_x_/sodium alginate (SA) aerogel with aligned channels prepared by this unidirectional freeze casting (UFC) method (Figure 6a) [52]. By UFC, the Ti_3_C_2_T_x_/SA mixed dispersion in a Teflon mold with an attachment of a copper base (cold finger) immersed in liquid nitrogen, the as-formed pyramid-like ice crystals exclude the SA-adhered Ti_3_C_2_T_x_ sheets, leading to the formation of a hybrid aerogel. SEM images show that the aerogel has oriented cell walls and unidirectional pore channels with gaps of tens of micrometers from the side view, and a honeycomb-like cellular structure from the top view.

#### 2.2.2. 3D Printing

Additive manufacturing technologies, which are capable of printing 3D objects, appear as a paradigm for scalable manufacturing porous monoliths [100,101]. Prior to the printing, understanding the rheological features of the material dispersions is necessary. Gogotsi and co-workers studied the rheological behavior of Ti_3_C_2_T_x_ aqueous dispersions, from single-layer to multiple-layer Ti_3_C_2_T_x_, from colloidal dispersions to high-loading slurries, and from viscous to viscoelastic properties [102]. They found a shear-thinning behavior of MXene dispersions, and the rheological properties could be effectively tuned by flake size selection, volume fraction/concentration of dispersion, and shearing rates. Processability charts for aqueous dispersions of Ti_3_C_2_T_x_ were provided, where the ratio of elastic moduli to viscous moduli is plotted as a function of frequency, superimposed with approximate regimes of operation techniques to see the change in rheology of the system at different processing rates. The charts showed great potential of MXenes in electrospraying, spray coating, ink-jet printing, wet spinning, extrusion printing, and dry spinning. With this knowledge in mind, Barg and co-workers modulated 3D printable inks based on large lateral size, few-layer thick Ti_3_C_2_T_x_ flakes aqueous suspension [103]. These inks presented a typical shear-thinning behavior that can easily flow through narrow nozzles and instantaneously recover to the solid state after being printed, enabling the extruded filaments to retain their shape, even in multiple-layered stacking. By using continuous extrusion-based 3D printing and freeze drying, a series of 3D MXene constructs, such as woodpile, hollow rectangular prism, and interdigitated electrode configurations, were achieved. Figure 6b shows that the lattice structure in the prepared woodpile is supported by the interlaced configuration of printed filaments with hundred micrometer channels, where each filament is 326 ± 13 µm in diameter, formed by a cross-assembly of Ti_3_C_2_T_x_ flakes.

A limitation of the above ink is the relatively high concentration of MXenes required by the ink rheology, however, leading to agglomeration. Recently, several groups addressed this challenge by developing the ink composition or printing process [104,105,106,107,108]. Sun and co-workers added a trace amount of a divalent cation, e.g.*,* Zn^2+^, into the Ti_3_C_2_ suspension, assisting ink gelation to satisfy the rheology demanded by 3D printing [105]. Qiu and co-workers reported a 3D printed template-assisted assembling approach, where a resin template is firstly printed then filled by MXene inks via a predesigned sprue [107]. NaOH solution was then used to etch the template and to crosslink Ti_3_C_2_T_x_ nanosheets, forming a 3D hydrogel network. Wang and co-workers formulated a mixed aqueous slurry composed of V_2_CT_x_ MXenes, GO, and carbon nanotubes (CNTs) as ink [108]. After printing, the as-prepared microgrid hydrogel was freeze dried to form an aerogel. Then, an annealing process was performed to convert GO into rGO, creating a strong mechanical scaffold to support MXenes.

#### 2.2.3. 3D Printing Combined UFC

Liang and co-workers combined 3D printing and unidirectional freezing, developing a facile strategy to prepare MXene-based hierarchical architectures with finest structure regulation (Figure 6c) [109]. They formulated a Ti_3_C_2_T_x_-based nanocomposite ink, then deposited it layer-by-layer onto oxygen plasma-treated polydimethylsiloxane (PDMS) by the extrusion-based 3D printing technique, to build a thick interdigitated construct. UFC and freeze drying were subsequently adopted to engineer and solidify the filament into a honeycomb-like microporous scaffold, with most cell walls being parallel to the temperature gradient, achieving the designing of pores at a micro-level.

### 2.3. Aligned Lamellar Structure

An aligned lamellar microstructure consists of vertically aligned, layered walls and channels, which can be further divided into local oriented, long-range ordered, and radial types according to the morphology and regularity of walls. The introduction of these microstructures into MXene monoliths can be achieved by using the directional freezing technique, which is unidirectional, bidirectional, and radial FC, corresponding to the above microstructure types.

#### 2.3.1. Local Oriented Lamellar Microstructure

**Figure 7:**
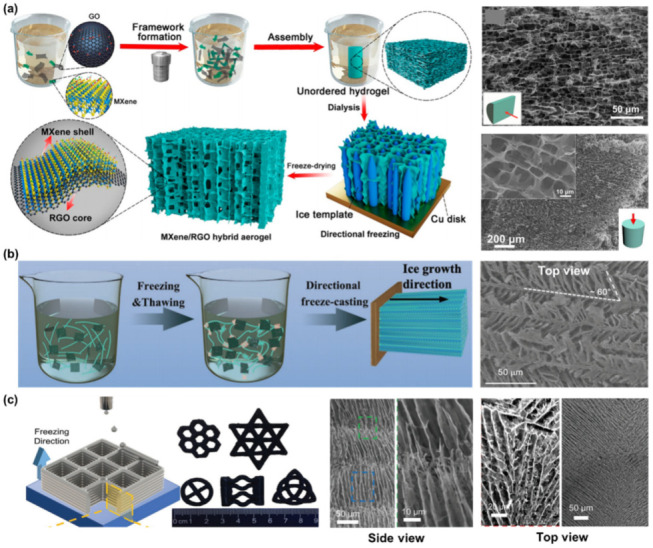
(**a**) Schematic illustration of MXene/rGO aerogel with aligned lamellar microstructure prepared by UFC, and SEM images captured from the side view and top view. (**b**) Schematic illustration of spectrally modified MXene/PVA aerogel with local oriented lamellar structure prepared by freezing/thawing, UFC, and freeze drying, and SEM image captured from the top view of this aerogel. (**c**) Schematic illustration of patterned MXene aerogel prepared by drop-on-demand inkjet printing and UFC, and SEM images captured from the side view and top view of this aerogel. (**a**) is reproduced with permission from ref. [49]. Copyright 2018, American Chemical Society. (**b**) is reproduced with permission from ref. [110]. Copyright 2022, Wiley-VCH. (**c**) is reproduced with permission from ref. [111]. Copyright 2021, Wiley-VCH.

Numerous short-ranged, interconnected building layers are characteristic of monoliths with a local oriented lamellar structure. Like the aligned honeycomb structure, it possesses penetrable channels in the longitudinal plane, yet, in the transverse plane, a series of relatively layered domains appear. MXene monoliths with this type of microstructure are commonly produced by UFC. Zhang and co-workers unidirectionally freeze-casted an unordered Ti_3_C_2_T_x_/rGO hybrid hydrogel to regulate its microstructure (Figure 7a) [49]. After freeze drying, a Ti_3_C_2_T_x_/rGO aerogel was obtained, where an aligned structure, formed by the guidance of vertical ice crystal, was observed in the side view, while, a layered morphology was seen in the top view.

Material design often draws guidance and inspiration from the natural world. Kim and co-workers, inspired by the structure of a penguin’s down feather, fabricated an MXene-based aerogel with a microstructure consisting of longitudinal struts connected by transverse ligaments (Figure 7b) [110]. They prepared a mixed suspension of spectrally modified Ti_3_C_2_T_x_/PVA and performed three iterations of freezing/thawing cycles to form a crosslinked hydrogel, then adopted UFC followed by freeze drying and heating to generate an aerogel. The addition of PVA and the repeated freezing–thawing process increase the viscosity of the freezing subject, inducing a dendritic ice growth (ice-structuring) during UFC, and thus, resulting in a unique feather-like microstructure, with main trunks along the temperature gradient and parallel ligaments at an angle of ≈60° from the struts.

Lin and co-workers reported a novel 3D freeze-printing method that combines drop-on-demand inkjet printing and UFC, to build aerogel [111]. Droplets of Ti_3_C_2_T_x_ ink were deposited onto a freezing substrate and quickly frozen once in contact. The 3D aerogels were fabricated through the layer-by-layer deposition of lines followed by freeze drying. Ti_3_C_2_T_x_ sheets in each layer were forced to align vertically and tightly, forming walls between the ice crystal boundaries. The top surface of this aerogel consists of randomly aligned lamellae, proving the local oriented lamellar feature (Figure 7c).

#### 2.3.2. Long-Range Ordered Lamellar Microstructure

**Figure 8:**
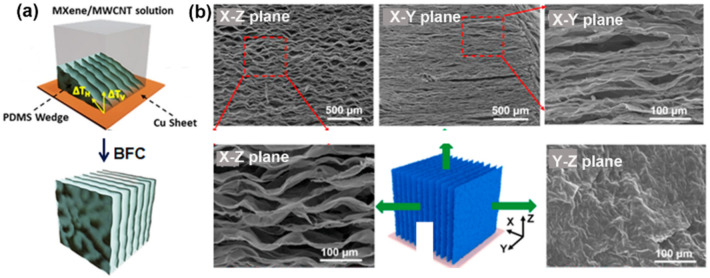
(**a**) Schematic illustration of BFC device and process. (**b**) SEM images of Ti_3_C_2_T_x_/PI aerogel with long-range ordered lamellar structure captured from different views. (**a**) is reproduced with permission from ref. [60]. Copyright 2019, American Chemical Society. (**b**) is reproduced with permission from ref. [54]. Copyright 2020, Elsevier.

Monoliths with this type of microstructure consist of uniformly arranged and large building layers. The formation of this morphology requires the generation of two orthogonal temperature gradients (horizontal and vertical) during freezing, which can be achieved by a bidirectional freeze casting (BFC) technique [99]. In general, BFC is performed in a thermal insulated mold, with a slope PDMS wedge at the bottom. When freezing, dual temperature gradients are generated simultaneously, resulting in a bidirectional growth of ice crystals and forcing the building blocks into an aligned lamellar arrangement (Figure 8a) [60]. Yin and co-workers [112], and other groups [60,113], used BFC to produce a series of MXene monoliths with a parallel lamellar structure. This anisotropic morphology was systematically characterized by Zhang and co-workers [54]. As shown in Figure 8b, the side views from the X-Z plane and X-Y plane show uniform lamellar structures with numerous bridges, while rough solid lamella surfaces are seen from the Y-Z plane.

#### 2.3.3. Radial Lamellar Microstructure

**Figure 9:**
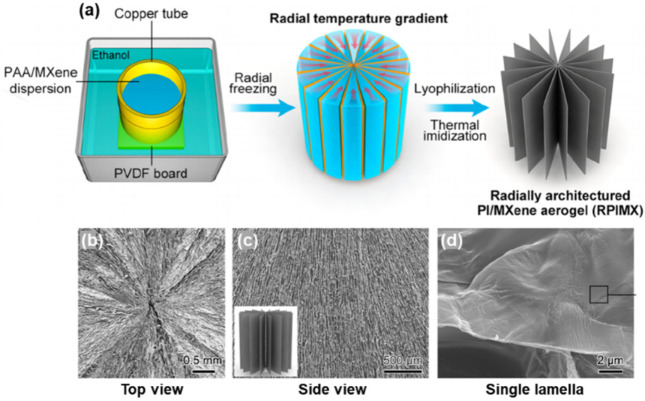
(**a**) Schematic illustration of RFC process and MXene/PI aerogel with radial lamellar structure. SEM images of the aerogel captured from the (**b**) top view and (**c**) side view, and (**d**) SEM image of a single lamella. Reproduced with permission from ref. [53]. Copyright 2022, American Chemical Society.

GO aerogel with a radial and centrosymmetric structure exhibited superior elasticity and absorption capacity to that of GO aerogels with “traditional” pore structures made by conventional FC, showing the great value of this configuration [114]. Building MXene monoliths with radially distributed lamellar structure is a cutting-edge topic, which was recently achieved by Liu and co-workers [53]. They reported a Ti_3_C_2_T_x_/PI aerogel with vertically and radially aligned layers formed via a radial freeze casting (RFC) process. The freezing mold is made by a copper outer wall with high thermal conductivity that can induce a growth of ice crystals from the periphery to the center and a polyvinylidene difluoride (PVDF) base that can prevent the vertical growth of ice. An obvious radial temperature gradient can be generated once the mold is immerged in liquid nitrogen (Figure 9a). The structural features are shown in SEM images from different views: in the top view, the Ti_3_C_2_T_x_/PI layers arrange in a well-defined centrosymmetric pattern (Figure 9b); the side view reveals a vertically aligned, ordered, layered structure (Figure 9c); and a zoomed-in image displays the 2D lamellar structure of a single layer (Figure 9d).

### 2.4. Microstructure Tailoring and Evaluation

It is notable that the property and function of monoliths are closely related to their microstructure. The ability to tailor the microstructure has a great impact on the mechanical strength, ion transport, and so on. The pore morphology and regularity of the monoliths constructed through the FC method depend on various intrinsic and extrinsic parameters, including the concentration and size of the solid loading, particle–particle interaction, particle–ice interaction, additives, external energized fields, and freezing rate/direction [115,116]. For MXene-based porous monoliths with an aligned microstructure, it has been reported that the concentration and freezing direction/rate can affect the porous structure, from the macroscopical level, e.g., regularity and spacing, to the finest level, such as sheet thickness and openings throughout the walls.

**Figure 10:**
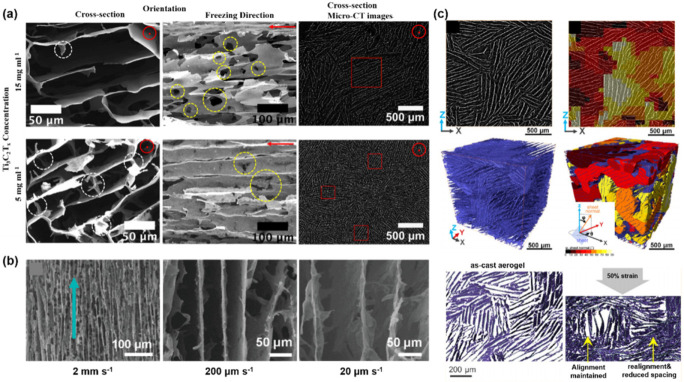
(**a**) SEM and Micro-CT images of MXene monoliths with local oriented lamellar structure prepared from suspensions with varying concentrations (15 and 50 mg mL^−1^). (**b**) SEM images showing the enlarged spacing of MXene hydrogels under reduced freezing rate. (**c**) Virtual CT section and 3D segmented rendering of the aerogel sheets (up left); virtual CT section and 3D segmented showing the domain orientations (up right); and microstructure evolution under compression (down). (**a**) is reproduced with permission from ref. [117]. Copyright 2019, American Chemical Society. (**b**) is reproduced with permission from ref. [118]. Copyright 2020, American Chemical Society. (**c**) is reproduced with permission from ref. [55]. Copyright 2022, American Chemical Society.

The concentration of colloids has an imperative effect on the growth of ice crystals and the morphology of subsequent monoliths. Gogotsi’s group [113] and Barg’s group [117] both found that, by increasing the MXene concentration, the interlayer spacing will shrink and synapses/bridges will form (Figure 10a), due to the less available space for the growth of ice crystals, and the trapping of MXene flakes within the ice crystals, respectively. It was also reported that the increase in MXene concentration could lead to fewer openings throughout the walls and smaller lamellae domains.

We have discussed that the freezing direction directly determines the structure of pores. On the other hand, the freezing rate can also strongly influence the final microstructure. Li and co-workers found that the widths of the aligned channels can be varied from ~20 to 200 μm by tuning the freezing rates from 2 mm s*^−^*^1^ to 20 μm s*^−^*^1^ (Figure 10b) [118]. Nyström and co-workers also claimed that the pore channels’ diameter can be influenced by the induced temperature gradient [59]. This phenomenon was explained by Saiz and co-workers, where a lower freezing rate gives a smaller ice front velocity, allowing colloidal particles to locally pin the ice front, leading to a transverse growth in ice crystals, and finally obtaining relatively wide channels within the porous monoliths after the ice sublimation [119].

Microstructural evolution during compression is of great importance in supercapacitors [117], sensors [120], and oil cleanup [121]. Visualizing this process is essential for understanding the in-service structural changes and optimizing material designing. Rawson and co-workers used synchrotron-source X-ray computed microtomography (CT) and built 3D images of the domains of Ti_3_C_2_T_x_ MXene aerogels with a local oriented lamellar structure (Figure 10c) [55]. By quantitatively analyzing the lamellar domains, sheet spacing, and sheet orientation, and tracking their evolution as a function of increased compression, they proposed domain collapse mechanisms for the lamellar structure under uniaxial compression: (1) compressive strain leads to a progressive realignment of sheets normal to the loading direction and a reduction in sheet spacing; (2) for domains that initially aligned with the loading direction, their orientation and sheet spacing are maintained until buckling. The analysis method presented is widely applicable, showing the capacity of quantifying fine nanoscale features in MXene monoliths with other types of microstructures, or monitoring the microstructure evolution in other applications.

## 3. Applications of MXene-based Porous Monoliths and the Roles of Porous Microstructures

MXenes with attributes of high electrical conductivity (up to 20,000 S cm^−1^) [13,122,123], excellent light-to-heat conversion efficiency (near 100%) [14], abundant surface groups, and notable mechanical strength [15,16,17,124,125], have emerged as muti-functional materials and show promise for physical, chemical, and environmental applications. Integrating MXenes into a porous framework can effectively address their aggregation and maximize the functions. Such porous microstructure possesses high inner surface areas and large cavities, imparting the MXene monoliths with preeminent performance in the fields of electrochemistry, EMI shielding/EMW absorption, and piezoresistive sensing, as well as, in turn, the accommodation of other functional materials. Moreover, those aligned pore channels, either honeycomb-like or lamellar, can give extra advantages, such as a super-elastic mechanical property and facilitated wave/mass transport, pushing MXene-based functional materials to a new level.

### 3.1. Isotropic Pores

#### 3.1.1. Energy Storage and Conversion

MXenes have high volumetric capacitance and highly reversible intercalation/deintercalation of ions [126]. MXene-based porous monoliths have been used as electrodes that show superior performance for energy storage [67,69,81,127] and capacitive deionization [36], due to the more exposed electroactive sites, shortened ion diffusion path for promoting redox kinetics, and a rich inner surface for ion storage. MXene aerogels can serve as Li nucleation sites for Li-metal batteries [63] or polysulfide reservoir for lithium-sulfur batteries [64], where the efficient adsorption interfaces and fast ion/electron transport given by the porous structure are of importance in their high efficiency.

#### 3.1.2. EMI Shielding and EMW Absorption

**Figure 11:**
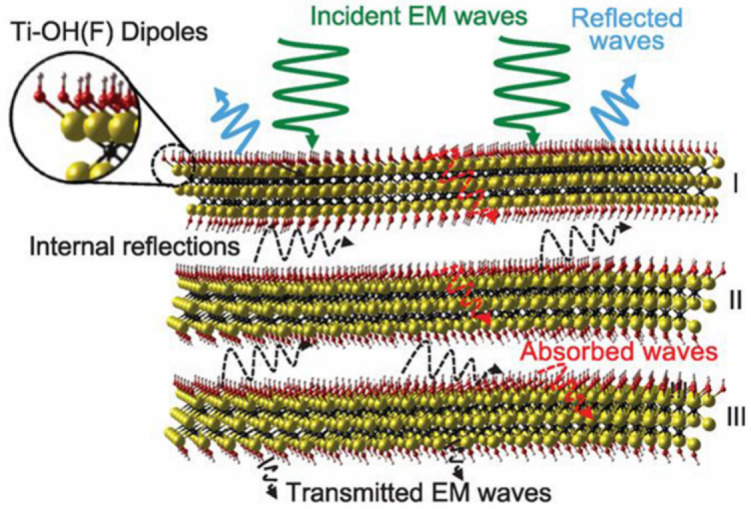
Schematic illustration of EMI shielding mechanism of MXene-SA composite films. Reproduced with permission from ref. [22]. Copyright 2016, the American Association for the Advancement of Science.

Modern electronic, wireless, detection and radar devices provide great convenience while simultaneously bringing a series of EM pollution to human life, which drives a fast exploration of advanced EMI shielding and EM absorption materials [128,129,130,131,132,133]. In 2016, Gogotsi and co-workers first reported a high EMI shielding Ti_3_C_2_T_x_-SA composite film and proposed mechanisms for its excellent performance (Figure 11) [22]. When an incoming EMW strikes the surface of film, the waves are partially reflected due to many charge carriers on the highly conductive surface. The remaining waves go through the MXene lattice, where interaction with the high electron density of MXene induces currents, contributing to ohmic losses and leading to a drop in energy. Local dipoles between Ti and terminating groups are generated when subjected to an alternating electromagnetic field, consuming the incoming EMWs by polarization losses. They also found that the interlayer space between the film layers gives rise to multiple internal reflections of the EMWs, facilitating the attenuation, and thus yielding an excellent EMI shielding capability. The 3D porous architectures with numerous pores have far more scattering centers for the internally reflected EMWs, greatly enlarging the intrinsic shielding ability of the materials [133]. Therefore, using MXene-based porous monoliths as EMI shielding or EMW absorption materials is one of the most effective strategies to manage the EM radiation (even terahertz) emission and interference [65,66,77,93,134].

#### 3.1.3. Wearable Piezoresistive Sensor

**Figure 12:**
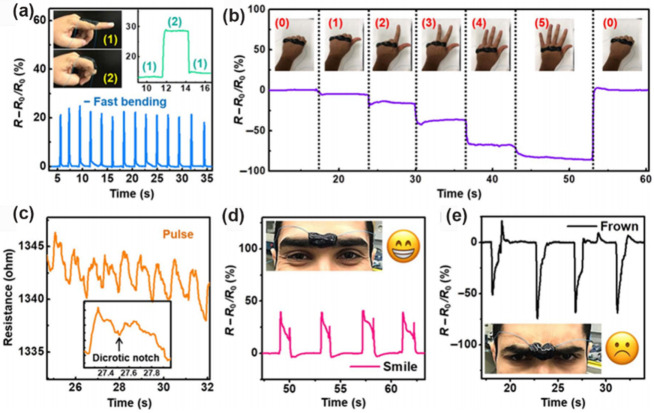
Resistance changes of MXene/PVA hydrogel in response to (**a**) finger bending, (**b**) different hand gestures, (**c**) human pulse, insert showing a typical dicrotic notch, and (**d**,**e**) facial expressions. Reproduced with permission from ref. [70]. Copyright 2018, the American Association for the Advancement of Science.

Advanced wearable electronics require strain sensors that are highly sensitive and stretchable, capable of adhering conformably to various surfaces. In virtue of the elastic property of polymer and the conductive property of MXenes, porous MXene/polymer monoliths hold great promise as sensors for wearable piezoresistive sensing application, where the geometric variation of the inner pores matters. Alshareef and co-workers characterized the geometry evaluation of the skeleton and the packing of Ti_3_C_2_T_x_ in a Ti_3_C_2_T_x_/PVA hydrogel under compressing [70]. The conductive resistance of the initial hydrogel is high, due to the large spacing between the Ti_3_C_2_T_x_ nanosheets. In contrast, when the hydrogel is compressed, the geometry becomes shorter, allowing Ti_3_C_2_T_x_ to have contact with each other and thus, resulting in a decrease in resistance and a corresponding increase in the current. Based on this mechanism, a series of advanced MXene/polymer monoliths serves as wearable sensing devices for real-time monitoring of small strains typically found in the human physiology (Figure 12a–e) [33,70,135,136]. Recently, Liu and co-workers developed a Ti_3_C_2_X MXene/PI nanofiber aerogel with typical “layer-strut” bracing hierarchical nanofibrous cellular structure [80]. Benefiting from the conductive skeleton and the robust connecting between MXene and PI nanofiber, a widely ranged detection of pressure was achieved, where a subtle compression (down to 0.5% compression, corresponding to 0.01 kPa) can cause the bend and contact of PI nanofibers, while the MXenes will contact when the compression strain increases to 60%. It is worth noting that the MXene/polymer piezoresistive devices can be imparted with versatile properties by using different functional polymers as components, such as strong anti-freezing [72], thermally stable [75], healable/degradable [72,79], or ammonia responsiveness [78], that can satisfy various application requirements. For instance, Shen and co-workers developed a multifunctional bio-aerogel based on a degradable bacterial cellulose/Ti_3_C_2_T_x_ hybrids with 3D porous structures that can be used for monitoring occlusal force and the release of NH_3_ from dental caries, offering a powerful platform for the preliminary screening of dental diseases [78].

#### 3.1.4. Accommodation

The inner pores and pore walls can serve as scaffolding to support functional materials, affording the opportunity to engineer superstructures for a variety of applications. Functionalization with Eosin Y (EY) photosensitizer [34], layered double hydroxides [137], CuO [138], Co particles [139], Pd [140], MOFs [141], Prussian blue [142], and Sb single atoms and quantum dots [143] allows the MXene hybrid monoliths to be used for photoredox catalysis, supercapacitors, acetone sensing, room-temperature Na-S batteries, catalytic hydrogenation of nitroaromatic compounds, alkali-ion batteries, real-time H_2_O_2_ monitors for living cells, and K^+^ batteries. The porous structure not only provides anchoring sites, but also facilitates application performance as compared to the restacked composites. For instance, Xu and co-workers demonstrated that the EY-functionalized Ti_3_C_2_T_x_/rGO hydrogel performs with higher photoredox catalytic efficiency than corresponding powders, which can be attributed to the interconnected porous structure that (1) endows the hydrogel with extended conductive networks and multidimensional electron transport pathways, to facilitate the separation and transfer of charge carriers produced from the visible light excitation of EY; and (2) has a large surface area, promoting the adsorption of reactants and thus enhancing the photoactivity. In addition, the framework offers a chance for drug loading and controllable release. Zhang and co-workers reported a Ti_3_C_2_/cellulose hydrogel with large pores that favors anticancer drug loading [71]. Due to the effective light adsorption and light–heat conversion of Ti_3_C_2_, the pore volume could be enlarged by a local temperature heating, enabling a light-triggered drug release.

### 3.2. Aligned Channels

#### 3.2.1. Anisotropic Mechanical Property and Wearable Piezoresistive Sensing

**Figure 13:**
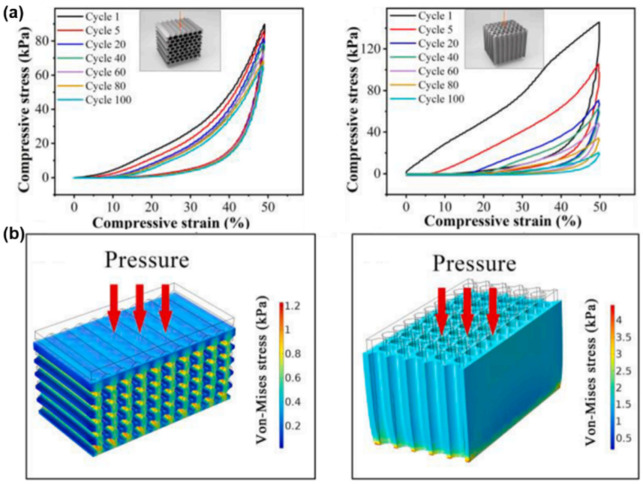
(**a**) Cyclic compressive stress–strain curves and (**b**) finite element analysis of MXene aerogel with aligned honeycomb structure. Reproduced with permission from ref. [145]. Copyright 2021, Elsevier.

The anisotropic structure imparts MXene monoliths with an anisotropic mechanical property. Li and co-workers used a compression test to demonstrate that the Ti_3_C_2_T_x_/gelatin hybrid aerogel with an aligned honeycomb structure has an orientation-dependent mechanical strength, where a high compression resilience only occurs in the direction that is normal to the aligned channels [144]. Zeng and co-workers fabricated a Ti_3_C_2_T_x_/carboxylated CNT/carboxymethyl chitosan hybrid aerogel and used it as a model to systematically investigate this oriented mechanical property [145]. As shown in Figure 13a, the stress–strain curves show good resilience when the external force is applied normally to the orientation direction, sharply contrasting with the other direction. Finite element analysis was performed to simulate the stress distribution of the aerogel compressed from two directions, and the obtained 3D finite element models at a strain of 50% are presented in Figure 13b. The aerogel model, under a perpendicular compression, shows a uniform von Mises stress distribution between 0.4 and 0.8 kPa and the maximum stress on the inner wall of the skeleton is about 1.2 kPa. However, a larger stress of the skeleton of about 2 kPa could cause the collapse of the skeleton during compression cycles, which reveals obvious anisotropic mechanical properties of the monoliths with an aligned honeycomb structure. As for Ti_3_C_2_T_x_ MXene monoliths with an aligned lamellar structure, they also have an anisotropic mechanical property, where the excellent elasticity and fatigue resistance only exist in the direction that is normal to the oriented channels [54].

**Figure 14:**
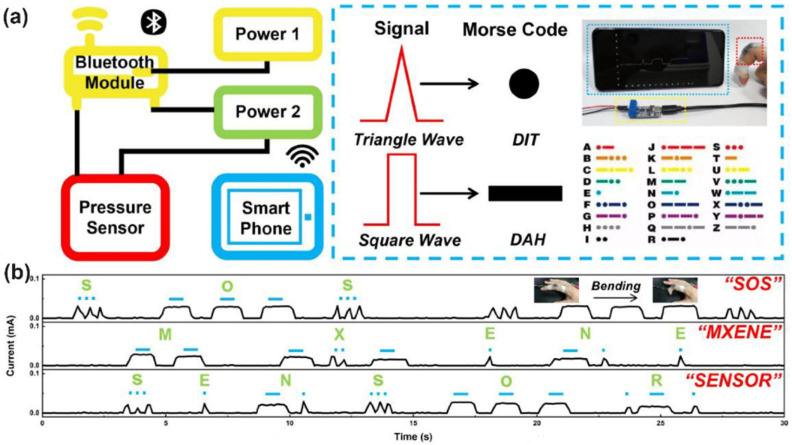
(**a**) Schematic diagram of the wireless device and signal codes. (**b**) Morse code signals represent different words. Reproduced with permission from ref. [148]. Copyright 2022, Elsevier.

Super-elastic MXene aerogels with an aligned microstructure hold great potential in wearable piezoresistive pressure sensing. Taking Ti_3_C_2_T_x_/rGO aerogels as examples, although the isotropic ones can be used for piezoresistive sensing, the elasticity (<80% of strain) still does not satisfy the expectations [120]. In contrast, aerogels with same components but with an aligned honeycomb microstructure can display a resilient strain of 95% [146]. The conducting and elastic skeleton provides fast electron transfer and stable structural integrity under compression/release cycles. When used for piezoresistive detecting, it performs with high sensitivity (0.28 kPa^−1^), wide detection range (up to 66.98 kPa), and ultralow detection limit (~60 Pa). Owing to the excellent flexibility, high sensitivity, and wide pressure range, several aligned MXene aerogel/hydrogel were derived, and wearable intelligent devices were developed for the real-time monitoring of human motions and health conditions [145,146,147]. More interestingly, by connecting the sensors with an external power and Bluetooth module to construct a signal generator, while using a smartphone as a signal receiver, the finger movement in Morse code can be wirelessly detected, achieving remote information transmission (Figure 14) [148].

**Figure 15:**
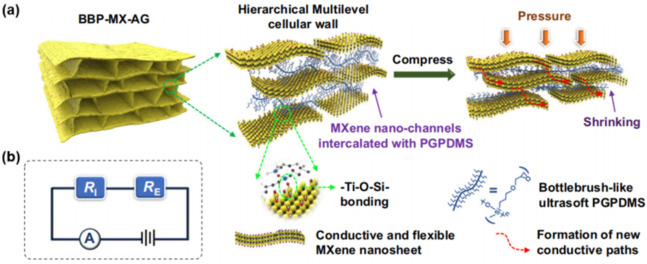
(**a**) Schematic illustration showing the MXene aerogel with sandwich-like structure, and the shrinking process of the multilevel cellular wall with bottlebrush-like PGPDMS crosslinked MXene nanochannels under compression. (**b**) Equivalent circuit diagram of this piezoresistive sensor. Reproduced with permission from ref. [149]. Copyright 2022, Springer Nature.

To further break the detection limits and expend the application conditions, Liang and co-workers proposed a new hierarchical structure, with multilevel cellular walls, for the Ti_3_C_2_T_x_ MXene aerogels. Typically, a “sandwich-like” design was proposed, where bottlebrush-like poly(3-glycidoxypropyldimethoxymethylsilane) (PGPDMS) with a soft polysiloxane main chain and flexible short side chains was intercalated into the aerogel walls (Figure 15a) [149]. The introduction of spacers not only prevents the restacking of Ti_3_C_2_T_x_ nanosheets, but also forms easily shrinkable nanochannels inside the cellular walls. The total resistance changes of aerogel in response to external forces are contributed to *R*_E_ and *R*_I_, in which *R*_E_ is external resistance changes induced by the bending or bucking of the cellular walls under compression, and *R*_I_ is the resistance changes induced by shrinking (or expanding) the spaces between nanochannels (Figure 15b). This aerogel has an ultralow Young’s modulus (~140 Pa at a density of 10 mg/cm^3^), remarkably reducing the critical stress value that triggers material deformation. When compressed, the multilevel nanochannels will shrink, promoting the contact of Ti_3_C_2_T_x_ nanosheets and thus, numerous new conductive paths form, and a considerable resistance change could be detected. Due to this ingenious design, the piezoresistive detection limit can be reduced to 0.0063 Pa with an ultrahigh sensitivity (>1900 kPa*^−^*^1^), which outperforms most reported pressure sensors. In another work, thermos-responsive semicrystalline poly(ethylene oxide) (PEO) was intercalated into the Ti_3_C_2_T_x_ MXene aerogel walls, generating a dual-sensing aerogel that can be used for simultaneously monitoring discriminable temperature and pressure [150]. When using as wearable device, it can detect the temperature-dependent characteristics of pulse pressure waveforms from artery vessels under different human body temperature states, which is promising for monitoring the real-time diagnosis of the human physiological state.

#### 3.2.2. Water Steam Generator/Solar Water Desalination

**Figure 16:**
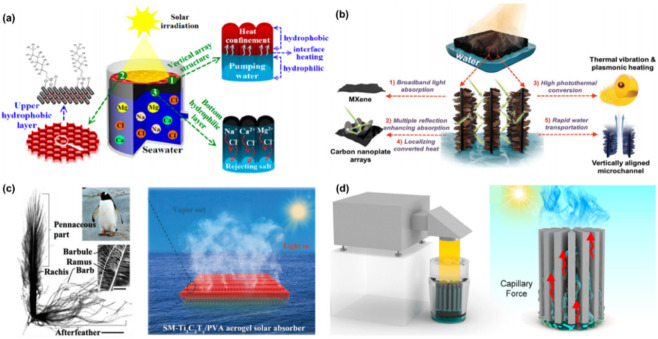
(**a**) Schematic illustration of a solar steam generation system and the salt resistance strategy based on a Janus-type MXene aerogel. (**b**) Schematic illustration of the solar-vapor generation from the solar absorber of MXene foam decorated with vertical arrays of 2D carbon nanoplates with embedded Co nanoparticles. (**c**) Photograph and optical microscope image showing a penguin down-feather (left); and schematic illustration of MXene/PVA aerogel with this microstructure for solar-powered water evaporation. (**d**) Schematic illustration of the radial MXene aerogel, photothermal evaporation device, and the capillary force in the aligned channels for water transportation. (**a**) is reproduced with permission from ref. [50]. Copyright 2019, American Chemical Society. (**b**) is reproduced with permission from ref. [160]. Copyright 2020, Wiley-VCH. (**c**) is reproduced with permission from ref. [110]. Copyright 2022, Wiley-VCH. (**d**) is reproduced with permission from ref. [53]. Copyright 2022, American Chemical Society.

Water scarcity is a global issue that drives the development of advanced desalination techniques for water harvesting [151]. Solar desalination is an effective method for seawater purification by converting solar energy to heat for vapor production [152,153], driving a fast exploration of high solar adsorption materials (such as carbon materials and black TiO_2_) and advanced architectures (such as hydrogel with many pores structure that can facilitate the water transport) [154,155,156]. The optimized combination of the materials, building constructions, and special structures can elevate light-absorbing and photothermal conversion efficiency. MXenes are considered ideal solar absorbers due to their nearly 100% light-to-thermal conversion and superior hydrophilicity. Perforated, oriented channels open light/water transport pathways to expand the application of MXene materials for solar water desalination/steam generation [53,106,118,157,158,159]. Quan and co-workers fabricated a vertically aligned Ti_3_C_2_ aerogel, then modified it to a Janus-type structure, where a hydrophobic upper layer can convert light to heat and lower the heat loss, while a hydrophilic bottom layer submerged in water can quickly pump water upward and enable the effective inhibition of salt crystallization due to the continuous pumping (Figure 16a) [50]. The vertically aligned channels facilitate capillary water transport, light absorption, and vapor escape, making this aerogel output a high conversion efficiency (87%) and a stable, long-term water yield (after 15 days) under 1 sun.

Based on the aligned microstructure, Liang and co-workers proposed a hierarchical design for the Ti_3_C_2_T_x_ foam, where the skeleton is decorated with vertical arrays of 2D carbon nanoplates with embedded Co nanoparticles to further increase the solar–vapor conversion efficiency (Figure 16b) [160]. It was found that the vertical arrays can: (1) create multiple light scattering and reflection, enhancing the solar light absorption across a broad spectrum of wavelengths; (2) reduce thermal conductivity; and (3) improve the chemical stability. These advantages, together with the rapid water transport and localized heating, impart the foam with up to ≈93.4% solar–vapor conversion efficiency for continuous water desalination. Very recently, Kim and co-workers were inspired by the excellent thermal isolating capability of penguin down feather, and they designed and fabricated a spectrally modified Ti_3_C_2_T_x_/PVA aerogel with similar microstructure (Figure 7b and Figure 16c), which contains longitudinal channels for water transport and transverse ligaments for suppressing the thermal loss, giving a high energy efficiency of 88.52% with an evaporation rate of 0.92 kg m^−2^ h^−1^ under a weak irradiation of 0.5 sunlight intensity [110]. Also enlightened by nature, Liu and co-workers produced a radial Ti_3_C_2_T_x_/PI composite aerogel, in which the microstructure is similar to the xylem parenchyma of dicotyledonous stems (Figure 9 and Figure 16d) [53]. The biomimetic radial water transporting channels impart the aerogel with excellent photothermal evaporation performance, with a water evaporation rate of 14.4 kg m^−2^ h^−1^ at solar irradiance of 4 sun.

#### 3.2.3. EMI Shielding and EMW Absorption

**Figure 17:**
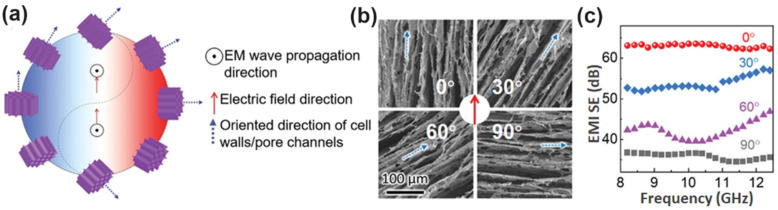
(**a**) Schematic illustration of the cell walls’/pore channels’ orientation-induced EMI shielding mechanism. (**b**) SEM images and (**c**) EMI shielding effectiveness of the MXene/CNF hybrid aerogels with various angles between the cell walls’ oriented direction and electric field direction of incident EM waves. Reproduced with permission from ref. [59]. Copyright 2020, Wiley-VCH.

Highly oriented cell walls govern EMI shielding performance, through an adjustment of their orientation angle to the electric field direction of the incident EMWs. Nyström and co-workers revealed that the EMI shielding effectiveness of the Ti_3_C_2_T_x_/CNF monolith was maximized when the internal channels are parallel to the electric field direction (Figure 17) [59]. Such a shielding tuning mode provides a wide range of controllable EMI performance without altering the frame materials, offering great chances for fabricating functional devices with aligned microstructures for excellent EMI shielding performance.

**Figure 18:**
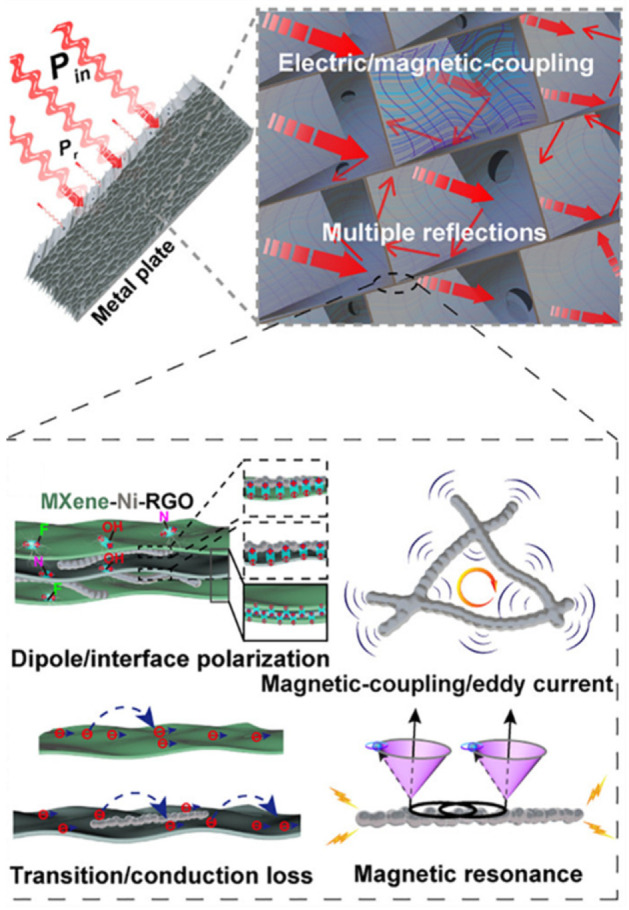
Schematic illustrations of the EM absorption mechanism of Ni/MXene/rGO aerogel. Reproduced with permission from ref. [167]. Copyright 2021, American Chemical Society.

A sophisticated microstructure design and a multicomponent strategy were adopted to 3D MXene monoliths to further optimize their EMI shielding and EMW absorption performance [52,144,161,162,163,164,165,166]. Xie and co-workers anchored magnetic Ni nanochains into an aligned Ti_3_C_2_T_x_/rGO aerogel [167], where the oriented channels and cell walls facilitate the entrance and the internal multiple scattering of EMW, respectively; the resulting 3D electric/magnetic-coupling network in the cell space effectively captured and attenuated the EMW (Figure 18). The cell walls consisting of Ni, Ti_3_C_2_T_x_, and rGO contribute to the synergistic dielectric losses (multiple heterogeneous interface polarizations, dipolar polarization, and conduction loss) and magnetic losses (magnetic resonance, magnetic-coupling effect, eddy current loss, etc.) of the incident EMWs. The hybrid aerogel exhibits exceptionally high EM absorption performance with a minimal reflection loss of -75.2 dB (99.999996% wave absorption) and a broadest EA band of 7.3 GHz. Very recently, Liu and co-workers reported a novel MXene sediment (Ti_3_C_2_T_x_ MXene and Ti_3_AlC_2_ MAX)/PVA/Ag nanowire (AgNW) hybrid hydrogel that has the best EMI shielding performance compared to the reported porous EMI shields [147]. This marked breakthrough is attributed to the synergistic efforts of multiple reflections, conductive loss, and polarization loss stemming from charge carriers of MXene sediment and AgNW, heterogeneous interfaces, and changed hydrogen bond networks associated with water molecules.

#### 3.2.4. Energy Storage and Conversion

In addition to the above applications, MXene monoliths with an aligned microstructure also show prospects in electrochemical fields, such as supercapacitors and batteries [105,107,108]. Yang and co-workers, as we mentioned in the Introduction, reported an excellent thickness-independent electrochemical performance based on a vertically aligned Ti_3_C_2_T_x_ electrode film for energy storage application [48]. Wu and co-workers prepared a vertically aligned Ti_3_C_2_T_x_ hydrogel treated by H_2_SO_4_ used as a capacitive electrode [168]. The synergistic effect of the intercalation of H^+^ and vertical alignment of Ti_3_C_2_T_x_ flakes enables sufficient ion diffusion and fast ion transport, respectively, rendering ultrahigh high capacitance (393 F g^–1^ at 5 mV s^–1^), and excellent rate capability (198 F g^–1^ at 1000 mV s^–1^). Gong and co-workers reported that a vertically aligned nanosheet arrays of Ti_3_C_2_T_x_ electrode, fabricated by ice-template-assisted blade coating, with an aligned and low tortuosity structure that could offer homogeneous and fast Li transport, is able to achieve a high Coulombic efficiency of 98.8% with more than 450 cycles at a fixed areal capacity of 1.0 mAh cm^−2^ at 1.0 mA cm^−2^ [169].

As for batteries, Lv and co-workers fabricated a lamellar aerogel with sandwich-like walls composed of Ti_3_C_2_T_x_/CNT [170]. Such a structure has an optimal physical blocking ability for the LiPSs, with enhanced exposure of Ti_3_C_2_T_x_ surface for the trapping and catalytic conversion, benefiting the confinement and rapid conversion of LiPSs, effectively suppressing the shuttling of LiPSs in high sulfur loading batteries. Sun and co-workers used vertically aligned Ti_3_C_2_T_x_/V_2_O_5_ aerogels as precursors to fabricate two thick electrodes that are vertically and horizontally aligned, respectively, by applying a mechanical press in two directions. The vertically aligned electrode delivers higher lithium-ion storage performance, due to the faster electron/ion transport over the entire electrode given by its vertical channels [171]. The summarized energy storage and conversion applications of MXene-based porous monoliths in different structural forms and their corresponding advantages are given in Table 1.

## 4. Conclusions

MXene-based porous monoliths have become an emerging class of advanced materials, where the inner pore structure affords a large interface area, rapid wave/mass transfer, and excellent mechanical properties for applications, including energy storage and conversion, EMI shielding/EMW absorption, solar/capacitive seawater desalination, and wearable piezoresistive sensors. Progress in the design of the pore structure has been markedly enhanced by the understanding and use of processing strategies. Gelation and templating with concentrated emulsion droplets were developed to generate foams, hydrogels, and aerogels with isotropic cellular structures, while anisotropic structures were able to be produced by FC and 3D printing techniques. Isotropic pore structures with a range of pore characteristics are easily produced and provide model systems for understanding the roles of porous microstructures in applications. Aligned channels yield anisotropic mechanical, structural, and physical properties, providing specific advantages for applications. A summary including the type of microstructures, fabrication methods, features and advantages, and applications of MXene-based porous monoliths is shown in Table 2.

We are still, though, in the early stages of the development of MXene-based porous monoliths, with numerous opportunities in the designing and manipulating of the pore structures and, hence, applications. For instance, incorporating 1D functional materials with MXenes at the liquid–liquid interface, such as Ni nanochain or Ag nanowire, may regulate the in-plane organization of MXene nanosheets, as well as enriching the components of the final architectures, imparting the emulsions and the subsequent aerogels with novel mechanical and physical properties. MXene-based porous monoliths with multilevel hierarchical architectures, such as those that are sandwich-like or penguin’s feather-like, have demonstrated great promise in piezoresistive sensing, batteries, and solar water desalination, inspiring more advanced designs for the microstructure. Introducing electrical- or magnetizable-responsible particles into MXene suspensions, then performing an external force field to regulate FC will likely help to guide the growth of ice crystals and thus, manipulate the microstructure of MXene monoliths. Coupled with these are developments in imaging and tomographic methods to quantitatively map out the 3D organization of the MXenes, correlate this organization with the processing conditions, and provide insights to exquisitely control the spatial distribution of the MXenes and, hence, their properties. The promise of MXene-based porous monoliths is significant, but it rests on developing a quantitative understanding of the science underpinning the processing to generate a specific structure so that design rules can be established to reproduce and tailor the structure.

## Figures and Tables

**Table 1:** Summary of MXene-based porous monoliths in energy storage and conversion.

Applications	Structural Forms	Advantages	Ref.
Supercapacitor electrode	Vertically aligned MXene electrodes; porous 3D MXene foam, monolith, aerogel, and hydrogel	Abundant active sites for ion storage, superb gravimetric capacitance and rate performance, outstanding cycling stability	[48,67,81,107,127,168]
Sodium-ion batteries	MXene monolith, V_2_CT_x_/rGO-CNT aerogel	Sufficient Na^+^ storage sites and multi-dimensional ion transport pathways	[69,108]
Li metal anodes	MXene aerogel, v-Ti_3_C_2_T_x_ electrodes, v-MXene/V_2_O_5_ electrode	Fast Li^+^ transport capability and abundant Li nucleation sites, high cycling stability and low overpotential, high gravimetric capacities and rate performance	[63,169,171]
Li-S batteries	3D porous MXene/rGO (MX/G) hybrid aerogel, PA-MXene/CNT aerogel	Low polarization, reduced interfacial impedance, and fast redox kinetics; ultra-stable cycling with a high sulfur loading	[64,170]
Zn-ion hybrid capacitors	3D-printed MXene cathode	Large interlayer spacing and Zn ions diffusion rate, excellent areal capacitance and rate capability	[105]

**Table 2:** Summary of MXene-based porous monoliths in terms of the type of microstructures, fabrication methods, features and advantages, and applications.

Type of Microstructures	Fabrication Methods	Features and Advantages	Applications	Ref.
Isotropic cellular	Assisted gelation Emulsion template	Facial and versatile fabrication process; tunable pore size and morphology; addressed restacking; conductive skeleton; high electron transfer rate and fast ion diffusion; multiple internal reflection; elastic MXene/polymer monoliths; offering accommodation for functional materials	Energy storage and conversion; EMI shielding and EMW absorption; wearable piezoresistive sensor; accommodation	[33,34,63,64,65,66,67,68,69,70,71,72,73,75,78,79,80,81,86,93,95,96,120]
Aligned honeycomb	UFC 3D printing 3D printing and UFC	UFC or 3D printing instruments requiring; tunable channel width; flexible macro- and microscopic structure designing; anisotropic structure and property; offering light/water transport pathways	EMI Shielding; solar water desalination; wearable piezoresistive sensor; energy storage and conversion	[52,59,103,105,106,107,108,118,144,145,146,148,149,150,157,167]
Local oriented lamellar	UFC	UFC instruments requiring; tunable domain size; anisotropic structure and property; offering light/water transport pathways	EMI Shielding; energy storage and conversion; solar water desalination	[49,55,110,111,117,147,160]
Long-range ordered lamellar	BFC	BFC instruments requiring; tunable interlayer width; anisotropic structure and property in two directions	EMI shielding and EMW absorption	[54,60,112,113,172]
Radial lamellar	RFC	RFC instruments requiring; elastic and conductive; offering light/water transport pathways	Wearable piezoresistive sensors; solar evaporators	[53]

## Data Availability

Not applicable.
